# Pleiotropic genes underlying genetic correlations across human diseases

**DOI:** 10.18699/vjgb-26-28

**Published:** 2026-04

**Authors:** I.V. Zorkoltseva, N.M. Belonogova, A.V. Kirichenko, Y.A. Tsepilov, T.I. Axenovich

**Affiliations:** Institute of Cytology and Genetics of the Siberian Branch of the Russian Academy of Sciences, Novosibirsk, Russia; Institute of Cytology and Genetics of the Siberian Branch of the Russian Academy of Sciences, Novosibirsk, Russia; Institute of Cytology and Genetics of the Siberian Branch of the Russian Academy of Sciences, Novosibirsk, Russia; Institute of Cytology and Genetics of the Siberian Branch of the Russian Academy of Sciences, Novosibirsk, Russia; Institute of Cytology and Genetics of the Siberian Branch of the Russian Academy of Sciences, Novosibirsk, Russia

**Keywords:** genetic correlation, common diseases, pleiotropic genes, gene-based association analysis, cross-trait meta-analysis, functional enrichment analysis, генетическая корреляция, распространенные болезни, плейотропные гены, анализ ассоциаций на уровне гена, метаанализ, биоинформатический анализ

## Abstract

Genetic correlation is a key characteristic of the global genetic similarity of human traits. Its primary underlying mechanism is pleiotropy, which operates at various biological levels. Gene-level pleiotropy is of particular interest, as genes are the fundamental functional units of the genome. Using publicly available results from genome-wide association studies for 324 diseases, we selected a set of 45 diseases in which every pair exhibited a significant genetic correlation. These diseases belonged to 10 nosological categories. The search for genes with pleiotropic effects was carried out using three approaches: (1) gene-based association analysis, (2) selection of single nucleotide polymorphisms (SNP) within gene coding regions significantly associated with at least two diseases, and (3) a cross-trait meta-analysis of SNP association signals followed by the identification of independent loci and gene prioritization within those loci. A comprehensive bioinformatic analysis was performed on all genes identified through these methods. We identified 167 pleiotropic genes implicated in 39 diseases. The most pleiotropic genes in our study were LPA, TCF7L2, SLC22A3, FES, CDKN2B, and APOE, which were associated with 7 to 9 diseases each. Bioinformatic analysis revealed that the pleiotropic genes identified for these 39 diseases are also involved in the genetic architecture of 501 other diseases and traits. This indicates a high degree of pleiotropy, facilitated by the involvement of these genes in diverse biological processes – including homeostasis, cell-cell signaling, regulation of cell proliferation, transport, and catalytic activity – and various molecular functions, such as signaling receptor binding. Thus, we demonstrated that 87% of diseases within a fully connected correlation network share associated genes with at least one other disease. This finding strongly suggests that genetic correlations between human diseases are largely driven by the pleiotropic effects of shared genes.

## Abstract (ru)

Характеристикой глобальной генетической общности признаков человека считается их генетическая корреляция. Ее основной механизм – плейотропия, проявляющаяся на различных уровнях. Наиболее интересна плейотропия на уровне генов, поскольку именно они являются фундаментальными функциональными единицами генома. Используя результаты полногеномного анализа ассоциаций 324 болезней, находящиеся в открытом доступе, мы отобрали группу из 45 болезней, в которой каждая пара показала значимую генетическую корреляцию. Эти болезни принадлежали 10 нозологическим категориям. Поиск генов с плейотропными эффектами осуществляли с помощью трех подходов: полногеномного анализа ассоциаций на уровне гена; выбора однонуклеотидных полиморфизмов (SNP) внутри кодирующей части гена, значимо ассоциированных хотя бы с двумя болезнями; и метаанализа сигналов ассоциации SNP со всеми болезнями с последующей идентификацией независимых локусов и приоритизации генов в них. Для всех отобранных таким образом генов мы провели биоинформатический анализ. Всего было идентифицировано 167 плейотропных генов, вовлеченных в контроль 39 болезней. Наиболее плейотропными в нашем исследовании были гены LPA, TCF7L2, SLC22A3, FES, CDKN2B и APOE, каждый из которых контролировал семь-девять болезней. Мы провели биоинформатический анализ всех генов и показали, что найденные нами для 39 болезней плейотропные гены участвуют в контроле еще 501 заболевания. Полученные данные указывают на высокую плейотропную способность этих генов, которая обеспечивается их участием в различных биологических процессах, таких как поддержание гомеостаза, межклеточная сигнализация, регуляция клеточной пролиферации, транспорт веществ и каталитическая активность, а также выполнением ими различных молекулярных функций, в частности связывания с сигнальными рецепторами. Таким образом, мы показали, что 87 % болезней, представляющих полносвязную группу, имеют общие гены с хотя бы еще одной болезнью. Это свидетельствует о том, что генетические корреляции между болезнями в значительной степени обусловлены плейотропными эффектами генов.

## Introduction

Traditionally, diseases have been studied in isolation, as independent entities. However, patients frequently present with multiple chronic conditions. This poses a significant challenge in medicine, as treatment strategies and prognosis are highly dependent on the presence of concomitant pathologies. Certain groups of diseases co-occur in patients more frequently than would be expected by chance, suggesting non-random associations. This phenomenon, known as comorbidity, is largely driven by the genetic similarity between diseases (Rzhetsky et al., 2007; Wang et al., 2017; Jia et al., 2023). Genetic sharing has been shown to account for 46 % of observed comorbidity (Dong et al., 2021).

The advancement of high-throughput genotyping techniques and the increasing availability of genome-wide association study (GWAS) results for a vast number of human diseases have enabled the investigation of their genetic sharing. The development of methods to estimate genetic correlations between traits has allowed for the assessment of global pairwise genetic similarities across human traits and diseases, leading to the creation of an atlas of genetic correlations (Bulik-Sullivan et al., 2015). It was discovered that almost every disease is genetically correlated with at least one other human disease or trait.

However, knowledge of genetic correlations alone is insufficient for understanding the mechanisms underlying genetic sharing, the primary mechanism of which is pleiotropy, manifesting at various biological levels. Widespread pleiotropy at the level of genetic variants has recently been demonstrated for human traits (Mackay, Anholt, 2024). An analysis of GWAS results for 116 complex traits identified 2,293 independent loci and revealed that in nearly all of these loci, the lead variants associated with one trait were also significant for at least one other trait (Qi et al., 2024).

Of particular interest is gene-level pleiotropy, as genes are the fundamental functional units of the genome. The vast majority of studies investigate the pleiotropic effects of genes on a limited set of pathologies belonging to one or two nosological categories. For instance, pleiotropy has been studied for cardiovascular (Song J. et al., 2024), psychiatric (Song Q. et al., 2025), and respiratory (Chen et al., 2024) diseases. A large group of studies comprises comparisons of a disease from one category with a set of diseases from another; for example, diabetes and cardiovascular (Adebekun et al., 2024) or gastrointestinal (Adewuyi et al., 2024) diseases, and post-traumatic stress disorder with a set of cardiovascular diseases (Shen et al., 2025). Some studies compare two sets of diseases from different categories, such as gastrointestinal and psychiatric disorders (Gong et al., 2023).

Although the findings of these studies are valuable for specialists working with specific pathologies, they do not allow for an assessment of the contribution of pleiotropy to the genetic sharing across a large number of diseases.

In this study, we estimate the contribution of pleiotropic genes to the genetic sharing among a large number of diseases from different nosological categories. To this end, we utilize publicly available GWAS summary statistics for 324 diseases from the UK Biobank cohort. From these, we select a subset of diseases in which every pair of diseases exhibits a significant genetic correlation. This subset will hereafter be referred to as the “fully connected” group.

## Materials and methods

**Fig. 1.**
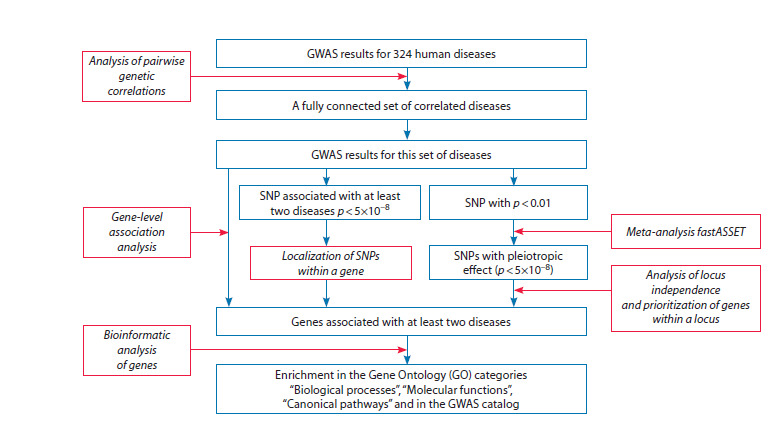
Study design. Data and results are indicated by blue boxes, while analysis methods are shown in red boxes and italicized type.

The study design is presented in Figure 1.

For 324 diseases, we estimated pairwise genetic correlations between them. A fully connected disease cluster was selected for further analysis. The analysis was performed using three approaches leveraging the GWAS results for these diseases. The first approach employs gene-level genome-wide association analysis to identify genes implicated in the etiology of at least two diseases. The second approach selects single-nucleotide polymorphisms (SNPs) significantly associated with at least two diseases and located within gene coding regions. The third approach is based on a meta-analysis of the association signals of each SNP across all diseases, followed by the identification of independent loci and prioritization of candidate genes within them. For all genes selected through these methods, we performed a bioinformatic analysis.

Materials. We utilized SAIGE summary statistics for UK Biobank phenotypes, derived from a sample of approximately 400,000 individuals of European ancestry (Zhou et al., 2018). These phenotypes corresponded to codes and nosologi- cal categories in accordance with the International Classification of Diseases PheCodes (Table S1 in Supplementary Materials)1.

Supplementary Materials are available in the online version of the paper: https://vavilov.elpub.ru/jour/manager/files/Suppl_Zork_Engl_30_2.xlsx


From 1,403 nosological disease codes, we selected those with more than 1,000 cases in the sample and excluded highly overlapping phenotypes. This resulted in a final set of 324 of the most common diseases. Summary statistics for each of these diseases were available for approximately 28 million single-nucleotide polymorphisms (SNPs).

The analysis included autosomal SNPs with a minor allele frequency (MAF) > 1×10−5. The major histocompatibility complex (MHC) region on chromosome 6 (positions 24 to 35 million base pairs) was excluded from the analysis.

For gene-level association analysis, we used matrices of genetic correlations between SNP genotypes within genes, which we had generated previously (Belonogova et al., 2022).

Analysis of genetic correlations. For the 324 selected diseases, we estimated genome-wide genetic correlations using the LDSC software (Linkage Disequilibrium Score Regression; Bulik-Sullivan et al., 2015). We identified a fully connected cluster of diseases where all pairwise genetic correlations were nominally significant (p ≤ 0.05).

Gene-level pleiotropy analysis. To identify genes with pleiotropic effects, we performed gene-based association analysis separately for each disease using summary statistics (z-scores and effect sizes) for each SNP and matrices of genetic correlations between all SNPs within a gene. The analysis included all SNPs located between the transcription start and end sites

The analysis was conducted using the sumSTAAR platform (Belonogova et al., 2022). We employed two summary statistics-based methods: SKAT-O (Liu D.J. et al., 2014) and PCA (Principal Component Analysis) (Wang, Abbott, 2008). These methods were implemented in the sumFREGAT package (Svishcheva et al., 2019). We used a weight function based on minor allele frequency (MAF), defined by a beta distribution with parameters (1, 1). For the PCA-based approach, we used the first principal components that explained at least 85 % of the total trait variance

The results from both analyses were combined using the ACAT-O method (Liu Y. et al., 2019). The analysis was restricted to protein-coding genes containing at least two SNPs with available summary statistics. The significance threshold was set at 2.5×10−6 (Bonferroni-corrected for 20,000 tests, 0.05/20,000).

Some genes were located within 500 kilobases of each other. For genes with an identical pattern of association signals (i. e., associated with the same set of diseases), we were unable to prioritize a single causal gene. However, to exclude false positive association signals within these gene clusters arising from linkage disequilibrium (LD), we utilized the Open Targets Platform database (Buniello et al., 2025). We excluded genes that showed no association (association score < 0.3) with any of the diseases common to the entire gene cluster

SNP-level pleiotropy analysis. In the first stage, we selected statistically significant associations from the GWAS data using a genome-wide significance threshold (p-value <5×10−8). For further analysis, we considered only those SNPs that were associated with at least two different diseases. To determine the functional role of significant SNPs, we used annotations from the Variant Effect Predictor (VEP, Ensembl) (McLaren et al., 2016). If a variant was located in a gene coding region (classified as a stop-gained, missense, or synonymous variant), the corresponding gene was considered associated with the diseases for which that SNP showed a significant association. Such genes were included in the list of potentially pleiotropic genes

Locus-level pleiotropy analysis consisted of two stages. In the first stage, we performed a meta-analysis of the association of each SNP with the full set of diseases using the fastASSET method (Fast Association Tests for Multiple Traits), implemented in the ASSET v.1.0.0 package (R/Bioconductor). In addition to assessing the significance of the pleiotropic effects for each SNP, this method identifies the specific subset of traits that contributes to the smallest meta-analysis p-value. To reduce computational time, the meta-analysis included only traits with a p-value below a specified threshold, and all traits were partitioned into relatively independent groups. The methodological foundation of this approach is detailed in (Bhattacharjee et al., 2012; Qi et al., 2024).

For the meta-analysis of each SNP, we selected diseases with a p-value < 0.1. A pleiotropic effect was considered significant at the genome-wide level if its meta-analysis p-value was <Subsequently, we identified independent genetic loci using the following algorithm to process the clumping results: if two index SNPs were located within 500 Kb of each other, their regions were merged, and the SNP with the smallest p-value was selected as the new index SNP. For SNPs with comparable significance (a difference in p-value of less than one order of magnitude), priority was given to the SNP located within a gene or the one associated with a larger number of traits. For SNPs within the same gene, the smallest p-value rule was applied. 5×10−8. We used the number of traits yielding significant p-values in the meta-analysis for a given SNP as a metric of its pleiotropy

In the second stage, we defined independent loci. First, we performed clumping using PLINK v1.9 (www.cog-geno mics.org/plink/1.9) with the parameters: ‘--clump-p1 5e-8 --clump-p2 1e-5 --clump-r2 0.1 --clump-kb 1000’. The analysis used linkage disequilibrium (LD) data from the 1000 Genomes Project (Phase 3, European population) and the coordinates of the reference genome GRCh37/hg19

Subsequently, we identified independent genetic loci using the following algorithm to process the clumping results: if two index SNPs were located within 500 Kb of each other, their regions were merged, and the SNP with the smallest p-value was selected as the new index SNP. For SNPs with comparable significance (a difference in p-value of less than one order of magnitude), priority was given to the SNP located within a gene or the one associated with a larger number of traits. For SNPs within the same gene, the smallest p-value rule was applied.

For each index SNP in the independent loci, we assigned the gene in which it was located or the nearest gene within the locus boundaries. This gene was considered pleiotropic

Bioinformatic analysis. Gene set enrichment analysis was performed using the GENE2FUNC module of the FUMA platform (Watanabe et al., 2017) (http://fuma.ctglab.nl/). All genes identified across the conducted analyses were used as the input gene list.

For the functional enrichment analysis of the identified genes, we utilized gene sets annotated in the Gene Ontology (GO) database (Biological Processes and Molecular Functions), canonical pathways, and the GWAS Catalog.

Default parameters were applied in all analyses. Multiple testing correction was performed using the Benjamini– Hochberg method. Results with an adjusted p-value <0.05 were considered statistically significant

## Results

**Analysis of genetic correlations**

**Fig. 2.**
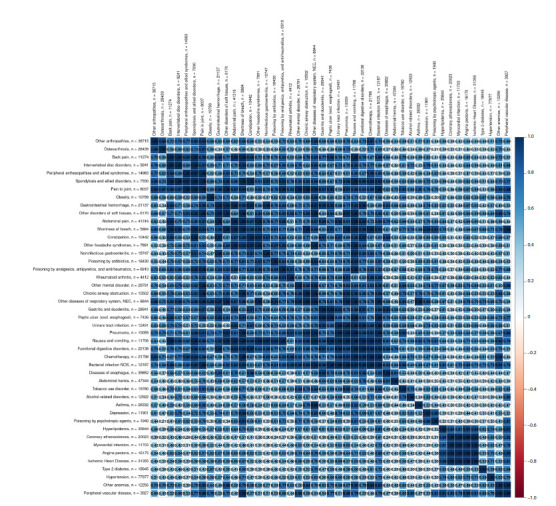
Heatmap of genetic correlations among 45 traits forming a fully connected cluster. n – the number of cases for each trait. The ‘hclust’ method was used for clustering.

Among the 324 diseases, 220 were genetically correlated with at least one other disease. However, only 45 of these diseases formed a fully connected cluster (Fig. 2).

**Fig. 3.**
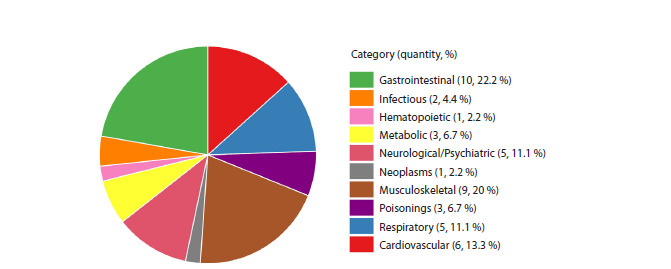
Distribution of the analyzed diseases across nosological categories.

These diseases represented ten nosological categories based on the PheCodes classification (Fig. 3). The list of diseases and categories is provided in Table S1. The most represented categories were gastrointestinal and musculoskeletal diseases.

**Gene-level pleiotropy analysis**

Significant gene-based association signals were identified for 26 out of the 45 diseases (Table S2). The largest number of associated genes was found in the categories of cardiovascular pathologies (400) and metabolic disorders (165). In total, 680 genes were associated with at least one disease. Among them, 129 genes (19 %) demonstrated an association with at least two diseases

We excluded eight genes that were in strong linkage disequilibrium with other genes and did not pass the filtering criteria based on the Open Targets Platform (see “Materials and methods” section). Consequently, 121 genes with pleiotropic effects were included in the subsequent analysis. These genes were implicated in the etiology of two to six diseases. The total number of diseases associated with at least one of the 121 genes was 19, belonging to six nosological categories (Table S3). The largest number of genes was shared between diseases of the cardiovascular category and metabolic disorders, as well as among diseases within these categories.

**Analysis of SNP-level pleiotropic effects**

From the GWAS results, we selected 1,389 SNPs significantly associated (p-value <5×10−8) with at least two traits. For each of these SNPs, we determined their location relative to genes. Thirty-six SNPs were located within the protein-coding regions of 23 genes. These genes were subsequently considered as candidate genes with potential pleiotropic effects. Shared genes were identified for 11 diseases from three nosological categories (Table S4).

**Analysis of locus-level pleiotropic effects**

Meta-analysis identified 3,210 SNPs significantly associated (p < 5×10−8) with two or more traits. The complete list of associations is presented in Table S5. Following clumping and filtration, 71 independent loci were identified (Table S6). The largest number of independent loci was observed on chromosomes 2 and 10 (9 and 7 loci, respectively). For 11 loci, no gene could be assigned to the index SNP. That is, out of 71 index SNPs, only 60 tagged genes. Shared genes were identified for 36 diseases from nine nosological categories.

**Comparison of results from different analyses**

**Fig. 4.**
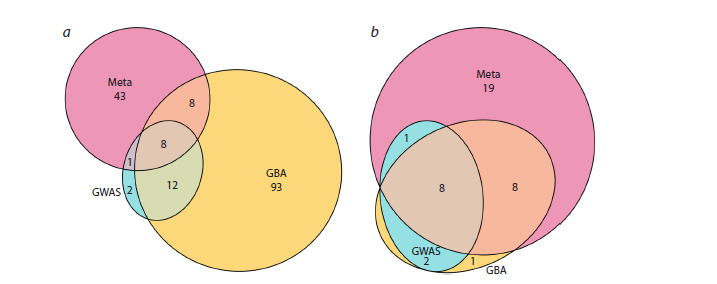
Diagram of the results from the three analytical methods (Gene-Based Analysis (GBA), Locus-Level (Meta), and SNP-Level (GWAS)). The circles represent: a – the number of genes identified by each method; b – the number of diseases sharing at least one gene with other diseases for each method.

Figure 4a presents a diagram illustrating the number of pleiotropic genes detected by each method and their overlap. As can be seen, the gene-level analysis identified the maximum number of pleiotropic genes, while the SNP-level analysis identified the minimum.

Figure 4b shows the distribution of the number of diseases for which at least one gene shared with other diseases was identified, depending on the method used. The results demonstrate that the locus-level analysis revealed the largest number of such diseases, while the SNP-level analysis revealed the smallest.

**Integrated analysis of results from all methods**

We combined the results obtained by all methods. It turned out that six diseases – poisoning by psychotropic agents, peripheral vascular disease, rheumatoid arthritis, shortness of breath, constipation, and chemotherapy – did not share genes with any other disease. The first four diseases had the lowest prevalence among the 45 selected diseases (Table S1). It ranged from 0.005 to 0.015. Prevalence was measured as the proportion of patients in a large population sample from the UK Biobank.

The distribution of the remaining 39 diseases across nosological categories was similar to the distribution of the original 45 diseases presented in Figure 3.

**Fig. 5.**
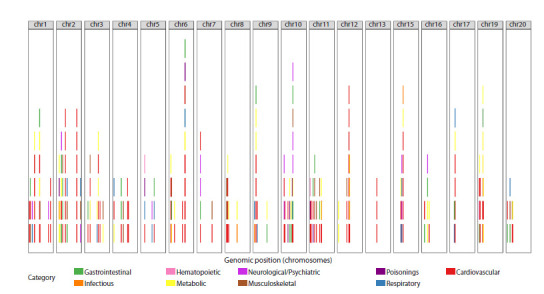
Genomic map of gene-disease associations. Each point corresponds to one disease, and its color represents the disease’s nosological category.

The total number of pleiotropic genes identified by all three methods combined was 167 (Table S7). Figure 5 shows the genomic distribution of these genes. As can be seen, they are located on all autosomes but with varying density. The number of diseases associated with each gene ranges from 2 to 9.

**Fig. 6.**
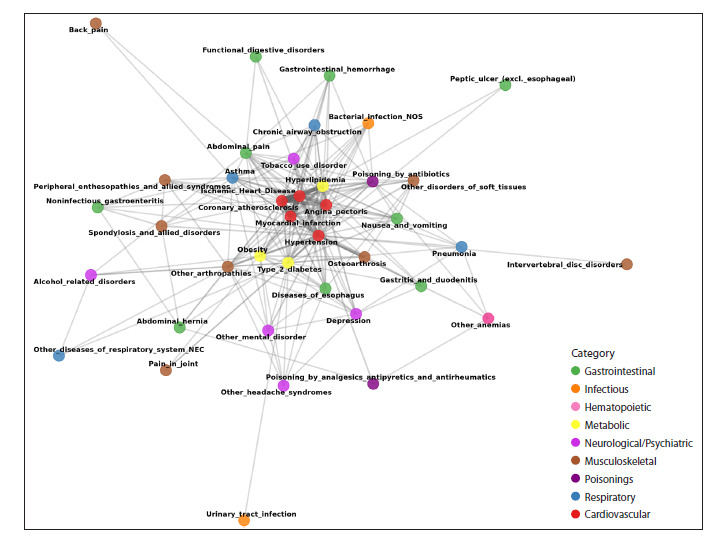
Network of diseases connected by shared genes. The color of the nodes corresponds to the disease’s nosological category. The edges (lines) represent genes shared by a pair of diseases, and the edge thickness corresponds to the number of shared genes.

We constructed a network of disease connections via pleiotropic genes (Fig. 6). As can be seen, diseases of the cardiovascular system and hyperlipidemia are most closely interconnected. All diseases except two (urinary tract infections and intervertebral disc disorders) are connected to at least two other diseases. Diseases belonging to the same category are, in most cases, directly connected to each other or linked via a single disease from another category. The majority of diseases exhibit multiple connections to other diseases, both within the same category and across different categories.

Pleiotropic genes are often categorized into those shared by diseases within a single nosological category and those, the pleiotropic effects of which span diseases across dif- ferent categories. Forty-four of the 167 pleiotropic genes were common only to diseases within a single category, while the remainder connected diseases from two to six categories. The most pleiotropic genes were LPA, TCF7L2, SLC22A3, FES, CDKN2B, and APOE, each associated with 7 to 9 diseases.

**Bioinformatic analysis**

We identified 261 statistically significantly enriched Gene Ontology (GO) categories: 252 categories in the “Biological Process” (BP) ontology and nine categories in the “Molecular Function” (MF) ontology. Among the enriched biological processes, the largest number of genes were associated with the regulation of homeostasis, response to endogenous stimuli, intercellular signaling, small molecule metabolic processes, regulation of cell proliferation, transport, and catalytic activity. Among the enriched molecular functions, the largest number of genes were assigned to the category “signaling receptor binding”. The enriched canonical pathways with the largest number of genes from our set were involved in “signaling receptor binding” and “transport of small molecules”. However, the canonical pathways demonstrating the highest statistical significance of enrichment were those related to lipid metabolism. These included: statin-mediated inhibition of cholesterol synthesis, cholesterol metabolism, plasma lipoprotein assembly, remodeling, and clearance, LDL, HDL, and triglyceride metabolic pathways (including associated diseases), plasma lipoprotein remodeling, and a pathway associated with familial hyperlipidemia type 2. Complete gene lists for the enriched biological processes, molecular functions, and canonical pathways are provided in Tables S8–S10.

Enrichment analysis using gene sets from the GWAS Catalog identified 501 significantly enriched traits. The most significant associations were observed with cardiovascular diseases (ischemic heart disease, myocardial infarction), blood pressure traits, lipid metabolism phenotypes, and body mass index (Table S11). This aligns well with the finding that the most significantly enriched canonical pathways are related to lipid metabolism.

## Discussion

In this study, we investigated the contribution of pleiotropic genes to the shared genetic basis of common human diseases. We examined a fully connected cluster of 45 diseases. The analysis included not only classic multifactorial diseases but also conditions, the development of which is largely associated with external factors. However, the very fact that these diseases exhibit significant genetic correlations with other diseases indicates the existence of a common genetic component. We showed that 39 of them (87 %) share genes with at least one other disease and can be represented as a connected graph (Fig. 6). For the remaining six diseases, we did not detect shared genes due to insufficient information about the diseases because of their low prevalence or the manifestation of pleiotropy at levels other than the gene level. This is supported by the fact that gene-level association analysis did not reveal a single significant gene for five of these diseases.

We employed three distinct approaches to identify pleiotropic genes, based on SNP-level, locus-level, and gene-level analyses. Collectively, these methods identified 167 genes with pleiotropic effects. A comparison of the approaches revealed that the largest number of pleiotropic genes was identified using gene-based association analysis. This method simultaneously leverages information from all SNPs within a gene during association testing. It is considered the most powerful approach for detecting associated genes, particularly when the analysis incorporates rare SNPs (Lee et al., 2012). The least powerful approach was the SNP-based analysis for identifying pleiotropic genes. However, this is likely less attributable to a fundamental limitation of the approach itself and more a consequence of the stringent gene annotation criteria we applied to SNPs to minimize false-positive results.

The genes we identified exhibited varying degrees of pleiotropy, with the number of associated diseases ranging from two to nine. The most pleiotropic genes were LPA, TCF7L2, SLC22A3, FES, CDKN2B, and APOE, each associated with 7–9 diseases. It is well-established that these genes play a key role in fundamental cellular and metabolic processes, which explains their broad influence on diverse phenotypes.

For instance, the TCF7L2 gene encodes a transcription factor that is a key component of the Wnt signaling pathway. This pathway regulates cell growth, motility, survival, and differentiation during development (Jin, Liu, 2008; Facchinello et al., 2017). The CDKN2B gene encodes a protein that acts as a cell growth regulator and controls the progression through the G1 phase of the cell cycle (Xia et al., 2021). The FES gene encodes a tyrosine kinase involved in regulating the actin cytoskeleton, microtubule assembly, as well as processes of cell adhesion and migration (Laurent et al., 2004). Disruption of such fundamental biological processes logically leads to an increased risk of developing a wide spectrum of diseases.

We performed a bioinformatic analysis of all 167 pleiotropic genes by assessing their representation in various functional annotations. The analysis revealed significant enrichment across multiple categories, indicating the broad functional involvement of these genes. Bioinformatic analysis showed that the identified pleiotropic genes are significantly enriched in associations with hundreds (501) of traits and diseases from the GWAS catalog, confirming their high pleiotropic potential. This universality is explained by the involvement of these genes in key biological processes, including the maintenance of homeostasis, intercellular signaling, regulation of cell proliferation, transport, and catalytic activity, as well as by their diverse molecular functions, such as signaling receptor binding.

Pleiotropic gene effects largely explain the phenomenon of comorbidity – the simultaneous development of several diseases in a single patient, which significantly complicates diagnosis and treatment (Gratten, Visscher, 2016). Identifying pleiotropic genes allows for the identification of new therapeutic targets and the development of drugs that simultaneously address multiple pathologies (Bao et al., 2024). Pleiotropy also opens the possibility of repurposing existing drugs for new diseases (Pushpakom et al., 2019). However, gene pleiotropy also complicates the development of targeted drugs, requiring the assessment of potential adverse effects on other diseases (Nguyen et al., 2019).

## Conclusion

In conclusion, we have demonstrated that 87 % of the diseases forming a fully connected cluster share genes with at least one other disease. Furthermore, all these diseases were integrated into a single connected network. The genes we identified influence diseases belonging to both single and multiple nosological categories. Collectively, these findings indicate that the genetic correlations between diseases are largely driven by the pleiotropic effects of genes

## Conflict of interest

The authors declare no conflict of interest.
